# Prognostic Factors of Survival in Patients with Peritoneal Metastasis from Colorectal Cancer

**DOI:** 10.3390/jcm11164922

**Published:** 2022-08-22

**Authors:** Fernando Mendoza-Moreno, Manuel Diez-Alonso, Belén Matías-García, Enrique Ovejero-Merino, Remedios Gómez-Sanz, Alma Blázquez-Martín, Ana Quiroga-Valcárcel, Cristina Vera-Mansilla, Raquel Molina, Alberto San-Juan, Silvestra Barrena-Blázquez, Miguel A. Ortega, Melchor Alvarez-Mon, Alberto Gutiérrez-Calvo

**Affiliations:** 1 Department of General and Digestive Surgery, General and Digestive Surgery, Príncipe de Asturias Teaching Hospital, 28805 Alcalá de Henares, Spain; 2 Oncology, Príncipe de Asturias Teaching Hospital, 28805 Alcalá de Henares, Spain; 3 Medicine and Medical Specialities, Faculty of Medicine and Health Sciences, University of Alcalá, 28801 Alcalá de Henares, Spain; 4 Ramón y Cajal Institute of Sanitary Research (IRYCIS), 28034 Madrid, Spain; 5 Immune System Diseases-Rheumatology and Internal Medicine Service, University Hospital Príncipe de Asturias (CIBEREHD), 28806 Alcalá de Henares, Spain

**Keywords:** colorectal cancer, cytoreductive surgery, hyperthermic intraperitoneal chemotherapy, synchronous peritoneal metastases, metachronous peritoneal metastases, peritoneal carcinomatosis, peritoneal metastases

## Abstract

Objectives: The aim of this study was to analyze the prognostic factors of survival in patients with peritoneal metastasis (PM) from colorectal cancer (CRC). The type of relationship between survival and the PM time of detection was used to determine whether it was synchronous with the primary tumor or metachronous. Patients and Methods: Retrospective observational study. It included patients treated for colorectal adenocarcinoma diagnosed between January 2005 and December 2019 who presented PM at the time of diagnosis or during follow-up. Variables, such as sex, age, differentiation grade, positive adenopathy (pN+), tumor size (pT), tumor location, mucinous component, peritoneal carcinomatosis index (PCI), and KRAS mutational status, were analyzed. Results: During the study period, 1882 patients were surgically treated for CRC in our hospital. Of these, 240 patients (12.8%) were included in the study after evidence of PM. The mean age was 67 ± 12 years (range: 32–92 years), and 114 patients were female (47.5%). The mean follow-up was 20 ± 13 months (median 12 months). The Kaplan–Meier survival at 36 months was higher in patients with metachronous PM (24% vs. 8%; *p* = 0.002), WT-KRAS tumors (31% vs. 15%; *p* < 0.001), N0 stage (30% vs. 19%; *p* < 0.001), T3 stage tumors (18% vs. 19% in T4A and 3% in T4B; *p* > 0.001), and tumors with classic adenocarcinoma histology (18% vs. 8%; *p* = 0.011). Patients with a PCI of 1–10 showed a likelihood of survival at 36 months of 56%, which was longer than that found in patients with a PCI of 11–20 (8%) or a PCI of >20 (0%) (*p* < 0.001). In the multiple regression analysis, the factors with an independent prognostic value were: poor grade of differentiation (HR 1.995; 95% CI: 1.294–3.077), KRAS mutation (HR 1.751; 95% CI: 1.188–2.581), PCI 11–20 (HR: 9.935; 95% CI: 5.204–18.966) and PCI > 20 (HR: 4.011; 95% CI: 2.291–7.023). Conclusions: PCI should continue as the as the most useful prognostic indicator in order to assess prognostic estimations as well as therapeutic and surgical decisions, but tumor grade and KRAS mutational status may help in the treatment decision process by providing complementary information. The time of PM detection did not achieve statistical significance in the multiple regression analysis.

## 1. Introduction

Colorectal carcinoma (CRC) is one of the main causes of cancer death in Western countries [1]. The incidence of CRC in 2020 was close to two million per 100,000 population [2]. CRC is the second most common malignancy in men after lung cancer and the third most common malignancy in women after breast and lung carcinoma [3]. Despite screening programs for colorectal carcinoma, consisting of a fecal occult blood test and subsequent colonoscopy, up to 25% of patients have metastasis at diagnosis, and 10–20% of patients will develop metastasis during follow-up [4,5]. The most frequent site of secondary tumor dissemination is the liver followed by the lung and peritoneal cavity. Near 4–8% of CRCs have peritoneal metastasis (PM) at the time of diagnosis (synchronous), and 20% develop metachronous PM during follow-up [6,7,8].

Until recently, the presence of PM in a patient with CRC has been considered an incurable situation, susceptible only to palliative treatment. The median survival time was 12 months with systemic chemotherapy and six months without it [7]. In recent years, there has been a substantial improvement in the prognosis of patients with metastatic CRC. The introduction of modern chemotherapy based on 5-fluorouracil + cisplatin/irinotecan [9,10,11] has changed the prognosis of survival. However, the survival of patients with PM remains poor, and lower than that of patients with metastatic spread out of peritoneal cavity [12].

Cytoreductive surgery (CRS) combined with hyperthermic intraperitoneal chemotherapy (HIPEC) has been introduced for the treatment of patients with PM. A significant improvement of five-year survival has been achieved for patients with PM [13]. However, this is a complex procedure with high morbidity, and appropriate patient selection for PM has not been clearly stated.

The identification of prognostic factors associated with OS is important for selecting and planning the treatment of cancer patients. Prognostic factors have been widely studied in CRC metastatic to the liver or lung, but only a few studies have investigated this matter in patients with PM [14,15]. It is known that the main determinant of survival in patients with PM is the grade of tumor burden present in the peritoneum, which is quantitatively defined by the peritoneal carcinomatosis index (PCI) [10]. However, the influence of other factors, such as pT stage, the presence of pN+ adenopathy, incomplete tumor resection, poor tumor differentiation or mucinous component, is not well understood. In a previous study of 149 patients, we found that patients with PM diagnosed at follow-up have higher overall survival (OS) than those with PM detected synchronously with the primary tumor [16]. However, other publications have reported higher OS for patients with PM synchronously detected with the primary tumor [17]. This discrepancy may be due to the type of patients included in each study.

The aim of the present study was to analyze the relationship of various clinical and histopathological factors with survival prognosis in patients with PM from CRC. The type of relationship between survival and the time of detection of PM was determined whether it was synchronous with the primary tumor or metachronous.

## 2. Materials and Methods

Retrospective observational study. Consecutive patients treated for colorectal adenocarcinoma between January 2005 and December 2019, who presented at the time of diagnosis or during follow-up PM were included. Patients were selected from the prospectively completed database of the Coloproctology Unit of the General and Digestive Surgery Department of the Príncipe de Asturias Teaching Hospital. The study was approved by the center’s ethics committee.

The main objective was to analyze the information on survival prognosis provided by various clinical and histopathological factors in patients with PM of CRC and to determine their relationship with OR survival. The concept of synchronous peritoneal metastasis (SPM) was established as that described at the time of diagnosis of colorectal carcinoma or within the first 6 months after initial diagnosis. Metachronous peritoneal metastasis (MPM) was metastasis detected during clinical follow-up at least 6 months after surgery.

### 2.1. Inclusion/Exclusion Criteria

The inclusion criteria were: age over 18 years; histopathology of the primary tumor compatible with colorectal adenocarcinoma; resection of the primary colorectal tumor; and presence of peritoneal metastasis detected intraoperatively or by imaging tests [MRI or CT scan with HIPEC protocol (administration of 20 mL water-soluble oral contrast (Gastrografin^®^) diluted in 200 mL of water].

The exclusion criteria were: Eastern Cooperative Oncology Group (ECOG) functional status >2; and presence of synchronous tumor of any other location or organ.

### 2.2. Variables

The diagnosis of PM of colorectal adenocarcinoma was made by histopathological confirmation of the lesion taken intraoperatively or by guided radiological puncture.

All patients were analyzed for demographic variables, such as sex and age, as well as variables derived from the primary tumor or its behavior (differentiation grade, presence of positive adenopathy (pN+), tumor size (pT), tumor location, mucinous component, presence of metastasis in another location) and medical treatment received. The peritoneal carcinomatosis index (PCI) [10] was calculated at the time of diagnosis of PM in each patient. KRAS mutational status (codons 12 and 13) was assessed in biopsies of tumor samples when the presence of metastasis was detected, and the results were categorized as KRAS wild type (WT-KRAS) or KRAS mutant type (MT-KRAS).

### 2.3. Treatment

After the diagnosis of PM, all patients were evaluated in a multidisciplinary medical committee that assessed the possible therapeutic options depending on the grade of extension, coexistence of metastasis in other organs, presence of local complications produced by the tumor and the functional status of the patient. Initial chemotherapy was followed by resective surgery, palliative surgery plus palliative chemotherapy, or symptomatic supportive care. Resection of the primary tumor was indicated, as a first measure, in cases of symptomatic tumors (obstruction, perforation or hemorrhage).

In patients who were considered candidates for resective surgery, chemotherapy programs with cisplatin/irinotecan/5-fluorouracil (FOLFOX/FOLFIRI) was administered in agreement with the Oncology Department. In cases with performance status (ECOG > 2) and without major comorbidities, bevacizumab or anti-epidermal growth factor (anti-EGFR) antibodies (cetuximab or panitumumab) were added, depending on KRAS mutation status. Six cycles were programmed. Tumor response was assessed by CT scan and/or MRI at the end of each cycle. The peritoneal disease response was quantified. In cases with PCI less than 10 and with absence of metastasis to other organs, cytoreduction surgery and HIPEC were indicated. In cases with poor or nonresponse, a second line of chemotherapy was established, or palliative symptomatic treatment was oriented according to the patient’s functional status. Accordingly, four lines of chemotherapy were described after the intervention (adjuvant chemotherapy, adjuvant chemotherapy + biological agent, palliative chemotherapy and HIPEC).

### 2.4. Statistical Analysis

The results were collected for the variables included in the study. For the present study, the PCI was classified into three categories as follows: PCI = 1–10, PCI = 11–20 and PCI > 20. The variables were input into a Microsoft Excel 2019 (v.19) (Microsoft, Redmond, WA, USA)spreadsheet. Statistical analysis was performed using SPSS (v.23) (IBM, Armonk, New York, NY, USA).

Follow-up was defined as the time elapsed between the diagnosis of peritoneal metastasis and the patient’s death or last contact. Initially, survival up to 36 months after diagnosis and median survival for each variable included in the present study were analyzed using the Kaplan-Meier estimator. In this study, overall survival (OS) was equivalent to cancer-related survival as in all cases that died it was caused by the CRC.

Next, the distribution of patient and tumor characteristics between the synchronous and metachronous PM groups was compared using the x-squared test.

Finally, the effect of each variable on survival was evaluated using Cox proportional hazard regression. The risk of death due to CRC was expressed by the hazard ratio (HR) and its 95% confidence interval. *p* < 0.05 was considered to indicate statistical significance.

## 3. Results

### 3.1. Patients and Characteristics

During the study period, 1882 patients were surgically treated for CRC in our hospital. Of these, 240 patients (12.8%) were included in the study after evidence of PM. The demographic and clinical characteristics of this group are shown in Table 1. The mean age was 67 ± 12 years (range: 32–92 years), and 110 patients were female (47.5%). The mean follow-up was 20 ± 13 months (median 12 months). The location of the primary tumor was in the right colon in 110 patients (45.8%), in the left colon in 101 patients (42.1%) and in the rectum in 29 patients (12.1%). Moreover, 81 patients (33.7%) had mutations in the KRAS gene. The peritoneal carcinomatosis index was between one and 10 in 51 patients (21.2%), between 11 and 20 in 101 patients (42%) and greater than 20 in 88 patients (36.6%). In 132 patients, the metastasis had a synchronous presentation (55%) with the primary tumor. In 96 patients (40%), the peritoneum was the only site of metastasis. Of the total patients, 106 (44.2%) patients received adjuvant chemotherapy together with biologic agents, 57 (23.7%) patients received 5FU-based chemotherapy only and 77 (32.1%) patients received symptomatic treatment only. In addition, 35 patients (14.6%) underwent debulking surgery and HIPEC.

### 3.2. Patient and Tumor Characteristics Categorized by SPM/MPM Time of Detection

The age of patients with SPM was higher than that of patients who developed MPM (70 ± 11 vs. 65 ± 12 years) (*p* = 0.002) (Table 1). Furthermore, SPM was more frequent in right colon cancers (52.2%; 69 patients) than in left colon cancers (44.4%; 48 patients) (*p* = 0.002).

Among primary tumors staged as pT4 (52 patients; 39.3%) as well as among those classified as pN2 (82 patients, 62%), synchronous PM was more frequent. In contrast, among pT3 tumors, MPM (65 patients; 60.1%) and N1 (51 patients; 47%) were more frequent (*p* > 0.001).

Tumors with mucinous histology (42 cases, 31.9%) (*p* = 0.014) as well as tumors with poor differentiation (46 cases, 35%) (*p* = 0.001) were more frequent among patients with SPM.

Comparison of PCI values demonstrated that patients with the presence of SPM had a higher PCI (>20) (57 patients, 43.1%), while those with MPM had intermediate PCI values (11–20) (46 patients, 42.5%) (*p* = 0.014).

Regarding adjuvant treatment, patients with SPM more frequently received palliative chemotherapy (49 patients, 37.1%) compared to those patients with MPM in whom the predominant treatment was chemotherapy together with a biological agent (59 patients, 54.9%) (*p* = 0.01).

No significant differences between the temporality of peritoneal metastasis (metachronous vs. synchronous) were found in relation to sex (*p* = 0.1), KRAS mutation (*p* = 0.212), metastasis type to extraperitoneal metastatic organs (*p* = 0.092) or HIPEC administration (*p* = 0.084).

### 3.3. Long-Term Survival

**Figure 1:**
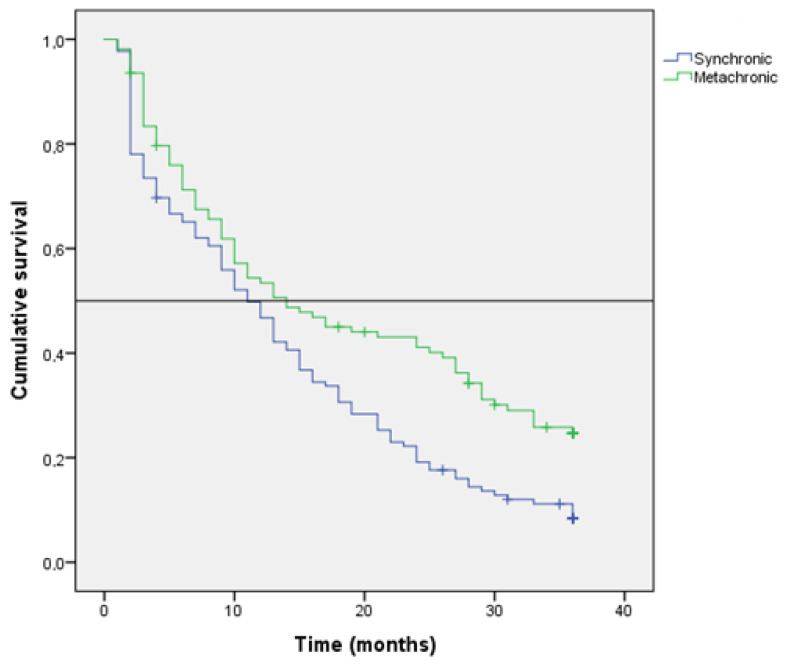
Kaplan-Meier survival function in the entire cohort according to the time of diagnosis of PM (Horizontal bar shows median survival).

**Figure 2:**
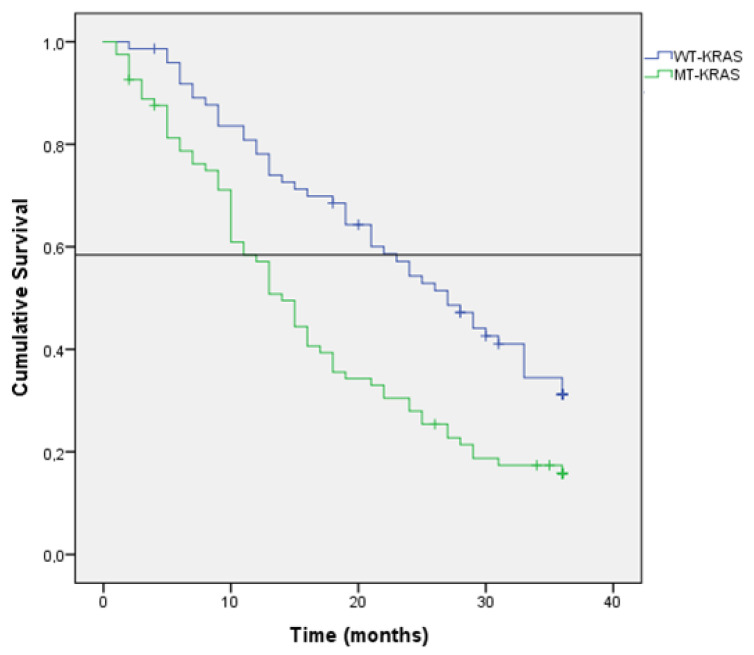
Kaplan-Meier survival in the entire cohort according to KRAS mutation status (Horizontal bar shows median survival).

**Figure 3:**
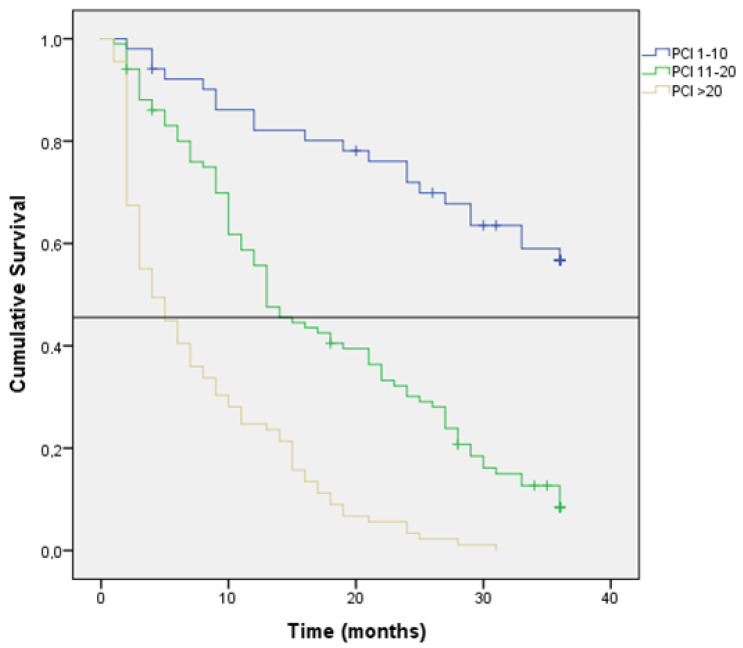
Kaplan-Meier survival curve according to PCI Index. Horizontal bar shows median survival.

Kaplan–Meir estimation of OS at 36 months after diagnosis was 32% (median: 21 months; 95% CI: 16–25). The results of the univariate survival analysis are shown in Table 2. The Kaplan-Meir’s estimation of survival at 36 months was higher in patients with MPM (24% vs. 8% (*p* = 0.002) (Figure 1), WT-KRAS tumors (31% vs. 15%; *p* < 0.001) (Figure 2), N0 stage (30% vs. 19%; *p* < 0.001), T3 stage tumors (18% vs. 19% in T4A and 3% in T4B; *p* > 0.001) and tumors with classic adenocarcinoma histology (18% vs. 8%; *p* = 0.011). Patients with a PCI of 1–10 showed a likelihood of survival at 36 months of 56%, which was longer than that found in patients with a PCI of 11–20 (8%) or with a PCI >20 (0%) (*p* < 0.001) (Figure 3). Additionally, the likelihood of survival was higher in patients who underwent HIPEC (64% vs. 7%; *p* < 0.001) and in patients who were treated with 5FU-based + bevacizumab/cetuximab chemotherapy programs (32% vs. 5%; *p* < 0.001).

Univariate analysis of survival (Table 2) indicated that the risk of dying was significantly higher in patients with pN2 tumors (HR, 3.69), tumors with mucinous histologic type (HR, 1.46), low grade (HR, 1.58), MT-KRAS tumors (HR, 1.88), and tumors with high PCI (HR, 12.78). The risk was lower in patients with metachronous PM (HR, 0.63).

### 3.4. KRAS Status Affects the Survival

**Figure 4:**
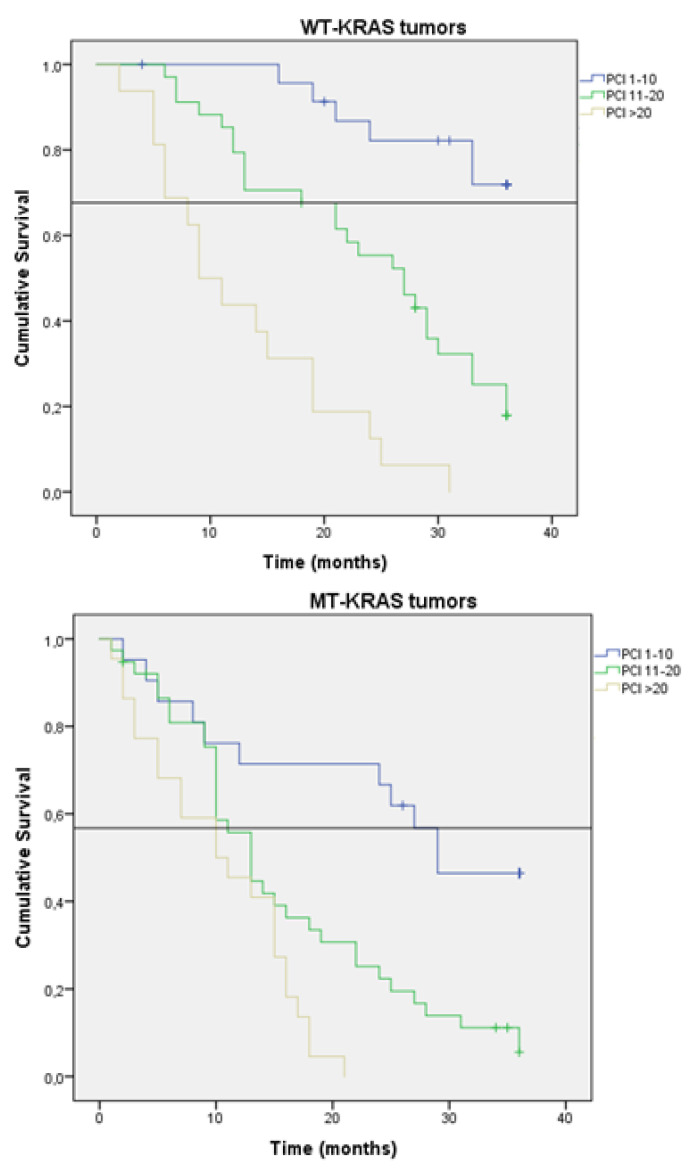
Kaplan-Meier survival function curve according to PCI Index and KRAS mutation status (Horizontal bar shows median survival).

In the three PCI categories, the OS varied according to KRAS gene status (Figure 4). In patients with a PCI of 1–10, survival at 36 months was 71% in patients with WT-KRAS tumors versus 41% in patients with MT-KRAS tumors (*p* = 0.025). In patients with a PCI of 11–20, those figures were 26% and 4% respectively (*p* < 0.001). In patients with a PCI >20, the survival at 36 months was nil, but at 28 months, the survival of patients with WT-KRAS tumors was 8% versus 0% for those with MT-KRAS tumors (*p* = 0.025). In the group of 36 patients treated with CRS/HIPEC, no difference in survival at 36 months was found between patients with WT-KRAS tumors and those with MT-KRAS tumors (*p* = 0.91).

Multiple regression analysis (Table 3) demonstrated that the factors with an independent prognostic value were poor grade of differentiation (HR 1.995; 95% CI: 1.294–3.077), KRAS mutation (HR 1.751; 95% CI: 1.188–2.581), PCI 11–20 (HR: 9.935; 95% CI: 5.204–18.966) and PCI >20 (HR: 4.011; 95% CI: 2.291–7.023).

## 4. Discussion

The peritoneal cavity is a frequent site for the development of metastasis in patients diagnosed with CRC. Although the presentation of peritoneal metastasis is different from that observed in the lung or liver, the established incidence for early diagnosis is estimated at 2–8%. This low incidence is due to the low sensitivity of imaging tests, such as computed tomography, for the detection of peritoneal metastasis (sensitivity of 43%), but the sensitivity has a direct relationship to size (sensitivity is 94% and 11% for lesions larger than 5 cm and smaller than 5 mm, respectively) [1].

It is estimated that approximately 6% of patients diagnosed with CRC have metastasis at the time of diagnosis. Of these patients, approximately 8% will have synchronous peritoneal involvement, while 20% will have liver metastasis [6,7,8,18]. The actual incidence of metachronous CRC metastasis is unknown but is estimated to be less than 10% based on a series. After analysis of the present data, we found that the incidence of synchronous peritoneal metastasis was 14.2%, while the incidence of metachronous metastasis was 5.7%.

The aims of this retrospective study were to analyze the differences between patients operated on for colorectal carcinoma with peritoneal metastasis in relation to the time of diagnosis (metachronous or synchronous) and to establish prognostic factors related to survival.

The development of MPM has been related to factors such as histological stage (T4, N2) or incomplete resection (R1/R2) after surgery of the primary tumor, bowel perforation or nonelective surgery [19,20]. However, in the present study, we observed significant differences in the development of MPM compared to SPM.

Younger patients (65 years) with intermediate stages (T3N1) and with an intermediate PCI (11–20) more frequently developed peritoneal metastasis during follow-up. This observation may be attributed to the fact that these patients had longer surveillance times and could make an early diagnosis of peritoneal metastasis in cases of recurrence. Moreover, the older patients in our series, who had a more advanced stage (T4 or N2) and higher PCI had a higher rate of peritoneal carcinomatosis at the time of the primary tumor intervention.

The prognostic value of the time of diagnosis of PM (Synchronous vs. Metachronous) have not been analyzed in detail previously. We found that OS at 36 months was higher in patients with metachronous PM (24% vs. 8% (*p* = 0.002). Only one study provided information with respect to this factor. Veld et al. found that patients with synchronous peritoneal metastasis showed lower survival than those with metachronous metastasis (18% versus 48% survival at 36 months) [13]. These results coincide with our data, but in addition, we found that this factor did not maintain prognostic significance in the multivariate analysis. We hypothesize that the worse evolution observed in Synchronous PM may be explained by the fact that many patients do not undergo surgery for the primary tumor or have a high PCI at diagnosis, whereas in many cases of metachronous metastasis the diagnosis is made by the clinical follow-up performed.

In agreement with Sánchez-Hidalgo et al., we observed a higher frequency of peritoneal metastasis of colorectal origin in relation to the location of the primary tumor, being more frequent in the colon (87.9%) than in the rectum (12.1%) [4]. Furthermore, Colloca GA et al. reported that the most frequent site for synchronous peritoneal metastasis is the right colon (32%), which was similar to our study (52.2%), and that metachronous peritoneal metastasis is more frequent in the descending colon [5].

A low grade of differentiation and the presence of a mucosal component were predictors of a worse prognosis. This association has been previously described with the addition of tumors with signet ring cells [20]. In the present study, patients with nonmucinous adenocarcinoma histology who had a good or moderate grade of differentiation had longer survival (18% and 19%, respectively; *p* = 0.01 and *p* = 0.00).

CRC patients staged as T4b (organ involvement) together with those with positive lymphadenopathy (pN2) have a worse prognosis with survival rates of 3% and 8%, respectively. In addition, a high PCI has been described as a relevant prognostic factor with lower PCIs having a better outcome (*p* = 0.00) [20].

The value of the KRAS oncogene mutation is an important prognostic factor in relation to survival in CRC patients [21]. Although KRAS mutation confers a status of greater resistance to chemotherapy treatment, patients who do not have the mutation (WT-KRAS) have a longer survival based on two principles as follows: (1) greater effect of chemotherapy; and (2) addition of a biological agent that causes a better tumor response. In our series, KRAS mutation determination was only determined in 154 patients (64.1%), and a longer survival was found for patients without KRAS oncogene mutation (12.9% vs. 6.2%; *p* = 0.001).

In contrast, Kammel Reid et al. [22] did not find an association between the development of peritoneal metastasis of CRC origin and KRAS oncogene mutation. Only a higher prevalence of mutated KRAS has been observed in CRC located in the ascending colon, which may be due to the higher prevalence of microsatellite instability in this location. In our series, although patients with the KRAS mutation had worse survival, there was no association with the location of the primary tumor.

We did not observe significant differences in survival in patients with peritoneal metastasis that progressed to other levels, such as the development of liver, lung or multiple metastasis, both in patients with synchronous metastasis and in those with metachronous metastasis. Similarly, other studies have reported similar survival for patients with peritoneal metastasis associated with extraperitoneal dissemination compared to those with only peritoneal involvement [23,24]. However, worse survival has been described in patients with unresected peritoneal metastasis than in patients with metachronous metastasis.

Currently, the development of new chemotherapy regimens and schedules associated with new biologic agents has changed the landscape in terms of survival. Chemotherapy regimens based on FOLFOX or FOLFIRI have reported five-year survival rates of 4% in patients with peritoneal carcinomatosis of colorectal origin [24]. In the present study, we observed increased survival in patients with peritoneal metastasis who had a biological agent added to the adjuvant chemotherapy schedule with a survival of up to 32% compared to conventional chemotherapy (5%) or palliative treatment (0%) (*p* = 0.00). This effect was related to patients with peritoneal metastasis who had WT-KRAS, which led to a greater tumor response after application of the biological agent.

Although we found no significant differences in relation to the number of patients who underwent cytoreductive surgery and HIPEC in our study, we observed a higher survival rate in patients who underwent cytoreductive surgery and HIPEC with the presence of peritoneal metastasis (64% vs. 7% *p* = 0.00) with a significant difference in relation to the type of peritoneal metastasis (metachronous 57.1%, synchronous 42.8%, *p* = 0.00). No significant differences were found in relation to the disease-free interval, which agreed with various studies reported in the literature [25].

However, patients with metachronous peritoneal metastasis are more frequently treated with cytoreductive surgery and HIPEC, which was observed in our series. Most likely, the longer survival in these patients suggests a less aggressive behavior when the metastasis appears during comprehensive clinical follow-up, but disease progression occurs concomitantly with the administration of systemic chemotherapy in most cases.

There are only a few studies that have analyzed the value of different prognostic factors in patients with PM [12,13,14]. The reason may be that the prognostic strength attained by the PCI is so high that it discourages investigators to analyze the influence of other prognostic factors. In this study, we have taken into consideration 13 clinical, histopathologic, and genetic variables in 240 patients and we have included patients with all grades of extent of the PM disease. To our knowledge, this is the largest study of prognostic factors in patients with PM from a single institution. Our analysis confirmed that PCI attain the highest relationship with survival and has strongest prognostic significance. In addition, we have observed that the KRAS mutational status and the tumor grade of differentiation have a relationship with survival in this type of patients and that these factors attain a prognostic information that is independent and complementary to that provided by the PCI. We conclude that PCI should continue as the as the most useful prognostic indicator in order to assess prognostic estimations, therapeutic and surgical decisions, but tumor grade and KRAS mutational status may help in the treatment decision process by providing complementary information. The time of PM detection did not achieve statistical significance in the multiple regression analysis 

## 5. Conclusions

The present study investigated a series of patients who underwent surgery for CRC with synchronous or metachronous development of peritoneal metastasis during follow-up. While patients with more advanced tumors (pT4 and pN2) or those with a higher PCI (>20/39) are more predisposed to the development of synchronous peritoneal metastasis, patients with intermediate stages (pT3N) or those with an intermediate PCI (11–20/39) more frequently present metachronous peritoneal metastasis. In relation to survival, the main prognostic factors are age, tumor stage (pTN), mucin presence, differentiation grade, Kras oncogene mutation, PCI, adjuvant chemotherapy treatment type, and performance of cytoreduction surgery and HIPEC.

## Figures and Tables

**Table 1:** Patients and tumor caracteristics.

	Synchronous Peritoneal Metastases (*n* = 132)	Metachronous Peritoneal Metastases (*n* = 108)	*p* Value
**SEX**			*p* = 0.106
Male	68	62	
Female	64	46	
**AGE**	70 ± 11	65 ± 12	*p* = 0.002
**LOCATION**			*p* = 0.020
Right Colon	69	41	
Left Colon	53	48	
Rectum	10	19	
**T STAGE**			*p* = 0.000
T3	40	65	
T4a	52	30	
T4b	40	13	
**HISTOLOGICAL TYPE**			*p* = 0.014
Adenocarcinoma	90	88	
Mucinous	42	20	
**N STAGE**			*p* = 0.000
N0	13	24	
N1	37	51	
N2	82	33	
**GRADE OF DIFERENTIATION**			*p* = 0.001
Well/Moderated	86	91	
Poor	46	17	
**KRAS STATUS**			*p* = 0.212
WT-KRAS	34	39	
MT KRAS	44	37	
**LOCATION OF METASTASES**			*p* = 0.092
Peritoneum (only)	52	44	
Peritoneum + Liver	47	24	
Peritoneum + Lung	5	6	
Multiple	28	34	
**PERITONEAL** **CARCINOMATOSIS INDEX**			*p* = 0.014
1–10	20	31	
11–20	55	46	
>20	57	31	
**CHEMOTHERAPY** **TREATMENT**			*p* = 0.010
5FU-Based Scheme + Biologic Agent	47	59	
5FU-Based Scheme	36	21	
Symptomatic	50	27	
**HIPEC**			*p* = 0.084
No	117	88	
Yes	15	20	

**Table 2:** Univariate analisis of survival.

	Patients (*n*)	Survival (Months)	*p* Value	HR	95% CI
**SEX**					
Male	126	14	*p* = 0.574	1.08	0.82–1.43
Female	114	16		1	
**DIAGNOSTIC OF** **METASTASES**					
Synchronic	132	8	*p* = 0.001	0.63	0.47–0.84
Metachronic	108	24		1	
**LOCATION**					
Right Colon	110	12	*p* = 0.183	1	
Left Colon	101	19		0.75	0.48–1.18
Rectum	29	14		0.78	0.58–1.05
**T STAGE**					
T3	105	18	*p* < 0.001	1	
T4a	82	19		1.89	1.33–2.71
T4b	53	3		1.07	0.77–1.49
**HISTOLOGICAL TYPE**					
Adenocarcinoma	178	18	*p* = 0.011	1	
Mucinous	62	8		1.46	1.75–2.00
**N STAGE**					
N0	37	30	*p* < 0.001	1	
N1	88	19		2.30	1.94–3.55
N2	115	8		1.39	0.88–2.19
**GRADE OF** **DIFERENTIATION**					
Well/Moderated	177	19	*p* = 0.002	1	
Poor	63	5		1.88	1.29–2.74
**KRAS STATUS**					
WT-KRAS	73	31	*p* = 0.001	1	
MT KRAS	81	15		1.88	1.29–2.74
**LOCATION OF** **METASTASES**					
Peritoneum (only)	96	20	*p* = 0.256	1	
Peritoneum + Liver	71	12		1.05	0.73–1.50
Peritoneum + Lung	11	9		1.38	0.95–1.99
Multiple	62	13		1.30	0.66–2.57
**PERITONEAL** **CARCINOMATOSIS INDEX**					
1–10	51	56	*p* < 0.001	1	
11–20	101	8		10.38	6.23–17.30
>20	88	0		2.40	2.40–6.33
**CHEMOTHERAPY** **TREATMENT**					
5FU-Based Scheme + Biologic Agent	106	32	*p* < 0.001	0.13	0.13–0.28
5FU-Based Scheme	57	5		0.81	0.54–0.12
Symptomatic	77	0		1	
**HIPEC**					
No	205	7	*p* < 0.001	1	
Yes	35	64		0.14	0.08–0.26

**Table 3:** Multivariate Survival Analysis.

	*p* Value	HR	CI 95% HR	
			Lower	Upper
**Poor Grade Differentiation**	0.002	1.995	1.294	3.077
**MT KRAS**	0.005	1.751	1.188	2.581
**PCI (1–10)**	0.000	1		
**PCI (11–20)**	0.000	9.935	5.204	18.966
**PCI (>20)**	0.000	4.011	2.291	7.023
**Syncrhonous/Metacrhnous**	0.358	1.219	0.799	1.859

## Data Availability

The data used to support the findings of the present study are available from the corresponding author upon request.

## Abbreviations

CCR	Colorectal Carcinoma
PM	Peritoneal Metastasis
HIPEC	Hyperthermic intraperitoneal Chemotherapy
CRS	Cytoreductiva Surgery
MPM	Metachronous peritoneal metastases
SPM	Synchronous peritoneal metastases
MRI	Magnetic Resonance Imaging
CT	Computed Tomography
ECOG	Eastern Cooperative Oncology Group
PCI	Peritoneal Carcinomatosis Index
WT-KRAS	Wild type KRAS
MT-KRAS	Mutant type KRAS
